# Astrocytic NLRP6 inflammasome: From protective sentinels to drivers of alcohol-induced neuroinflammation

**DOI:** 10.4103/NRR.NRR-D-24-01620

**Published:** 2025-07-05

**Authors:** Seema Singh, Shilpa Buch, Palsamy Periyasamy

**Affiliations:** Department of Pharmacology and Experimental Neuroscience, University of Nebraska Medical Center, Omaha, NE, USA

**Figure 1:**
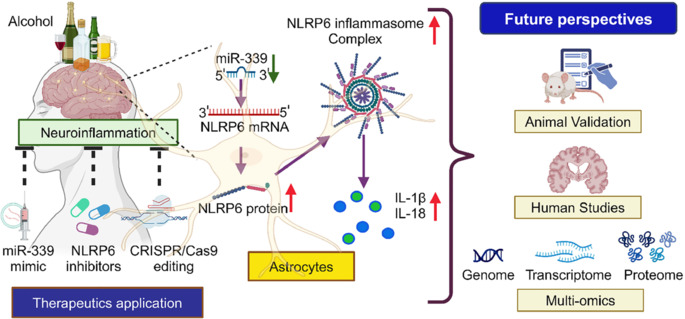
The miR-339-mediated NLRP6 inflammasome signaling in astrocytes and the ensuing neuroinflammation. Schematic illustrates various therapeutic applications, including miR-339 mimics, NLRP6 inhibitors, CRISPR/Cas9 editing, and future perspectives such as animal validation, human studies, and multiomics approaches (genome, transcriptome, and proteome). Created with BioRender.com. CRISPR-Cas9: Clustered regularly interspaced short palindromic repeats-associated protein 9; IL-18: interleukin 18; IL-1β: interleukin 1β; miR: microRNA; mRNA: messenger RNA; NLRP6: nucleotide-binding domain, leucine-rich–containing family, pyrin domain–containing-6.

The innate immune system of the central nervous system (CNS), long viewed as primarily microglia-driven, is now increasingly recognized to include astrocytes as active participants in neuroimmune signaling. Chronic alcohol exposure triggers oxidative stress, glial activation, and sustained inflammation, ultimately contributing to cognitive decline and neuronal injury. While microglial inflammasomes, particularly nucleotide-binding domain, leucine-rich–containing family, pyrin domain–containing-3 (NLRP3), have garnered attention in alcohol-related neuroinflammation, the recent study by Singh et al. (2025) extends this paradigm by identifying a miR-339-regulated NLRP6 inflammasome response in human fetal astrocytes exposed to ethanol. Their findings shed light on a potential astrocyte-specific inflammatory mechanism, but also raise key questions about its translational applicability (**Figure 1**). Specifically, the use of proliferating fetal astrocytes as a model system may better reflect mechanisms relevant to fetal alcohol spectrum disorders than adult alcohol use disorder (AUD). Furthermore, the focus on a single inflammasome axis overlooks the complex interplay among multiple innate immune sensors and their divergent roles across CNS cell types and species. In this Perspective, we critically examine the implications of the miR-339/NLRP6 axis in the broader landscape of astrocytic inflammasome research, discuss the limitations of the current model system, and highlight future directions for establishing NLRP6 as a viable therapeutic target for alcohol-induced neuroinflammation.

Chronic alcohol exposure perturbs the tightly regulated homeostasis of the CNS, fostering a sustained proinflammatory environment that drives neuropathological changes. Neuroinflammation, marked by glial activation and elevated proinflammatory cytokines, is a core feature of AUD, contributing to cognitive deficits and neuronal injury. While microglia have historically been the central focus of alcohol-induced inflammation, emerging research highlights a pivotal role for astrocytes in these processes. Astrocytes, responsible for neurotransmitter recycling, metabolic buffering, and blood–brain barrier maintenance, are particularly susceptible to ethanol-induced dysfunction (Jin et al., 2021). Singh et al. (2025) report that ethanol suppresses miR-339 in astrocytes, resulting in the derepression and activation of the NLRP6 inflammasome, with subsequent release of interleukin (IL)-1β and IL-18. This astrocyte-specific inflammasome signaling represents a significant shift in our understanding of neuroimmune mechanisms in AUD.

Interestingly, astrocytes appear to be enriched in NLRP6 relative to other inflammasomes such as NLRP3, further emphasizing their distinct immunological signature. While NLRP6 has been best characterized in peripheral organs, particularly the gut and liver, its CNS role is only beginning to be understood. In the intestine, NLRP6 helps maintain microbial and epithelial homeostasis and has been shown to modulate responses to colorectal cancer and alcoholic liver disease. Mouse models of alcoholic liver disease demonstrate that NLRP6 inhibition modestly disrupts intestinal balance without significantly altering liver injury (Mainz et al., 2022), while NLRP6 overexpression in alcoholic hepatitis suppresses hepatic inflammation and fibrosis through inhibition of nuclear factor κB signaling (Ji et al., 2020). In the CNS, Wang et al. (2017) showed that astrocytic NLRP6 expression following intracerebral hemorrhage exerted protective effects by attenuating brain damage. However, subsequent studies have reported that NLRP6 may contribute to inflammation and exacerbate neuronal injury in certain contexts (Zhang et al., 2020). These contrasting roles suggest that the NLRP6 function is highly context- and cell-type dependent.

Singh et al. (2025) extend NLRP6 investigation into CNS astrocytes under ethanol exposure, revealing proinflammatory activation rather than the protective effects observed in other models. However, their findings are based on human fetal astrocytes, a commonly used, though imperfect, model system. While fetal and adult astrocytes both express key astrocyte activation markers, i.e., glial fibrillary acidic protein and nuclear factor-I, and contribute to CNS homeostasis, immune regulation, and synaptic support, fetal astrocytes are more proliferative, easier to culture, and ethically more accessible. These properties make them valuable for modeling both fetal alcohol syndrome and aspects of adult AUD, particularly when adult astrocytes are difficult to obtain or culture. Still, given known differences in transcriptomic maturity and inflammatory responsiveness between fetal and adult glia, caution is warranted when extrapolating these *in vitro* findings to human adult pathology. Future studies using adult-derived cells or *in vivo* validation are essential to confirm whether ethanol-induced NLRP6 activation in astrocytes reflects a generalized mechanism in alcohol-related brain injury.

Inflammasomes are multiprotein complexes that detect pathogen-associated molecular patterns and damage-associated molecular patterns, initiating inflammatory signaling cascades. Their activation typically results in caspase-1 cleavage, leading to the maturation and secretion of IL-1β and IL-18, two central mediators of neuroinflammation. Among the inflammasome sensors, NLRP3 has received the most attention in CNS research due to its involvement in various neurodegenerative diseases, including Alzheimer’s disease, Parkinson’s disease, and multiple sclerosis. However, astrocytes also express other inflammasomes, including NLRP2 and AIM2, whose activation varies depending on disease stage and stimulus. For instance, NLRP3 is involved in experimental autoimmune encephalomyelitis, while AIM2 activation occurs during the later stages of experimental autoimmune encephalomyelitis. Both NLRP2 and NLRP3 have also been linked to lipopolysaccharide-induced depression-like behavior in murine models. In this context, the findings by Singh et al. (2025) identifying NLRP6 as a central mediator of ethanol-induced inflammasome signaling in astrocytes add a new dimension to our understanding of astrocyte-specific inflammatory responses. Their study shows that NLRP6 activation in human primary astrocytes is both dose- and time-dependent, with 50 mM ethanol eliciting the most robust response. Importantly, the study demonstrates that this NLRP6 activation occurs independently of other inflammasomes, such as NLRP3 and AIM2. This selective responsiveness underscores the unique functional role of NLRP6 in astrocytes and suggests that ethanol triggers a cell-type and sensor-specific inflammasome response, distinct from classical microglial NLRP3 activation.

These results also reinforce that astrocytes are not merely passive support cells but can undergo pathological transformation under chronic stress. Under physiological conditions, astrocytes regulate ionic balance, detoxify reactive oxygen species, support synaptic transmission, and maintain blood–brain barrier integrity. Chronic alcohol exposure, however, shifts astrocytes toward a reactive phenotype characterized by hypertrophy, cytokine release, and neurotoxicity. Singh et al. (2025) demonstrate that activation of the NLRP6 inflammasome promotes IL-1β and IL-18 secretion, not only perpetuating astrocytic reactivity but also stimulating microglial recruitment and activation. This astrocyte–microglia cross-talk exacerbates neuronal injury, disrupts synaptic function, and likely contributes to the progressive cognitive decline observed in AUD. While the identification of NLRP6 as a novel inflammatory driver in astrocytes opens potential therapeutic avenues, the regulatory mechanisms controlling its expression remain poorly understood. Currently, only a few suppressors of NLRP6 activity have been identified. Histamine and spermine have been shown to reduce NLRP6-mediated IL-18 secretion (Levy et al., 2015), although spermine binds with relatively low affinity, as demonstrated in structural studies by Shen et al. (2021). These sparse findings highlight the need for further research into the upstream modulators of NLRP6 in CNS cells and their context-specific effects in diseases like AUD.

MicroRNAs (miRs) are short, non-coding RNAs that function as post-transcriptional regulators of gene expression by binding to target mRNAs and promoting their degradation or translational repression. These epigenetic modulators play pivotal roles in maintaining cellular homeostasis and shaping immune responses under physiological and pathological conditions. Dysregulation of miRs has been linked to a wide range of diseases, including cancer, cardiovascular disease, and neurodegenerative disorders (Sarkar et al., 2019). Singh et al. (2025) identified miR-339 as a key regulator of astrocytic NLRP6 expression under ethanol exposure. Under basal conditions, miR-339 binds to the 3’-untranslated region of Nlrp6 mRNA, repressing its translation and thereby suppressing inflammasome activation. Ethanol exposure leads to downregulation of miR-339, releasing this inhibition and resulting in robust NLRP6 activation. This, in turn, promotes the secretion of IL-1β and IL-18, amplifying neuroinflammation. Restoration of miR-339 levels attenuates NLRP6 activation and cytokine release, suggesting a protective, regulatory role for miR-339 in astrocytic immune homeostasis (Singh et al., 2025). Interestingly, the function of miR-339 may be context-dependent, influenced by species, cell type, and inflammatory milieu. A study by Zhang et al. (2014) reported that alcohol exposure induces the transcription of miR-339, along with IL-6, IL-1β, and tumor necrosis factor α, in mouse brain tissue and isolated microglial cells. This response was mediated via nuclear factor κB activation, indicating that miR-339 may act downstream of or in feedback with canonical inflammatory transcription factors. The contrasting regulatory behavior of miR-339 across astrocytes and microglia suggests a cell-specific role in inflammation, warranting further investigation into its differential expression and function.

In the broader landscape of inflammasome regulation, there is emerging evidence from intestinal studies that NLRP6 acts as a negative regulator of excessive NLRP3 activation, helping to maintain mucosal immune balance. Whether similar crosstalk occurs in astrocytes remains unclear, but Singh et al. (2025) observed that ethanol did not alter NLRP3 levels in astrocytes, further supporting the unique and independent activation of NLRP6 in this context. It is plausible that miR-339 plays a dual regulatory role, fine-tuning both NLRP6 and NLRP3 inflammasomes to maintain immune equilibrium in tissue-specific ways. In the brain, where inflammasome signaling can drive both protective and pathological outcomes, understanding this regulatory axis may reveal new therapeutic targets not just for AUD, but for other neuroinflammatory and neurodegenerative conditions. These findings thus establish miR-339 as a promising therapeutic target for controlling astrocytic activation and mitigating alcohol-induced neuroinflammation.

The identification of the miR-339/NLRP6 axis in astrocytes introduces a promising yet complex therapeutic target for mitigating alcohol-induced neuroinflammation. The data from Singh et al. (2025) suggest that restoring miR-339 levels can suppress astrocytic NLRP6 activation and downstream IL1β and IL18 secretion. This highlights the potential of miR-based therapies for rebalancing neuroimmune homeostasis. However, translating such findings into clinical applications requires careful consideration of several unresolved challenges. First, miR delivery to the CNS remains technically difficult. Although lipid nanoparticles and other nanocarriers show promise, achieving cell-type specificity, especially targeting astrocytes without affecting other CNS cells, remains a major hurdle. Additionally, inflammasome modulation through small-molecule inhibitors or biologics must be approached with caution. NLRP6, like many inflammasome sensors, exhibits context-dependent functions: while proinflammatory in some CNS conditions, it has shown protective effects in others, such as limiting nuclear factor κB signaling in the liver and maintaining microbial balance in the gut (Wang et al., 2017; Ji et al., 2020). This duality highlights the potential risk of broad NLRP6 inhibition without cell-specific targeting.

Moreover, astrocytes themselves are functionally heterogeneous across brain regions and disease states. Interventions aimed at modulating astrocytic inflammasomes must account for this diversity. Even with gene-editing tools like CRISPR/Cas9, long-term safety, delivery specificity, and ethical considerations pose significant barriers to clinical use in neurological diseases. Adjunctive approaches may offer more immediate translational potential. For example, combining miR-339 restoration with currently approved AUD medications (e.g., naltrexone, acamprosate) could potentially enhance treatment efficacy by addressing both addictive behavior and underlying neuroinflammation. Co-administration with antioxidants might also help neutralize ethanol-induced oxidative stress while dampening inflammasome activation, offering a synergistic neuroprotective effect. Beyond AUD, NLRP6 and miR-339 may have broader relevance in other neurodegenerative and neuroinflammatory disorders. Shared pathological mechanisms such as glial activation, cytokine dysregulation, and autophagy dysfunction are common to neurodegenerative diseases such as Alzheimer’s disease, Parkinson’s disease, multiple sclerosis, and Huntington’s disease. NLRP6 has also been linked to lysosomal and autophagic pathways in peripheral organs, suggesting it may function as a broader immunometabolic sensor in the CNS. Multi-omic studies and region-specific models of astrocyte activation are needed to determine whether NLRP6 represents a convergent therapeutic target across CNS diseases or whether its modulation must be tailored to individual pathologies.

Despite its promise, targeting the miR-339/NLRP6 axis presents several translational challenges: (a) Delivery systems: Effective delivery of miRNA-based therapies or inflammasome inhibitors to the CNS is a significant hurdle. Advances in nanotechnology and blood–brain barrier-penetrating delivery systems will be critical. (b) Off-target effects: miRNA therapies must be carefully designed to minimize unintended interactions with non-target mRNAs, which could result in adverse effects. (c) Interindividual variability: Genetic and epigenetic differences may influence responses to miR-339-based therapies, necessitating personalized approaches. (d) Regulatory barriers: miRNA-based therapeutics face unique regulatory challenges, including stringent safety requirements and long-term efficacy assessments. (e) miR specificity: It is well recognized that one single miR can bind to several targets, and similarly, each target can be regulated by many miRs. Identifying the family of miRs and/or long non-coding RNAs regulating miR families will be critical for developing efficacious therapeutics.

Building on the findings of Singh et al. (2025), future research should prioritize (**Figure 1**): (a) *In vivo* validation: Translating these findings to animal models and human studies will be critical for establishing clinical relevance. (b) Mechanistic exploration: Investigating the upstream regulators of miR-339 and its potential crosstalk between NLRP6 and other inflammasomes will provide deeper insights into CNS inflammatory networks. (c) Multi-omic approaches: Integrating transcriptomic, epigenomic, and proteomic data could provide a comprehensive view of NLRP6-related pathways. (d) Long-term impact studies: Assessing the effects of chronic NLRP6 activation on synaptic plasticity, cognition, and behavior will be crucial for understanding its therapeutic potential.

While the study by Singh et al. (2025) provides valuable insights, it also raises important questions. For instance, how does NLRP6 activation in astrocytes influence neighboring neurons and microglia? What are the long-term effects of chronic NLRP6 activation on brain plasticity and cognition? Addressing these questions will be crucial for translating these findings into therapeutic applications. Another area of interest is the potential crosstalk between NLRP6 and other inflammasomes. Understanding how these pathways converge or diverge in different cell types could provide insights into the broader inflammasome network in the CNS.

The identification of the miR-339/NLRP6 inflammasome axis by Singh et al. (2025) represents a breakthrough in understanding alcohol-induced neuroinflammation. By linking ethanol exposure to astrocytic activation, the findings provide critical insights into the molecular mechanisms underlying AUD and highlight the development of novel therapeutic targets. Future research should explore the translational potential of targeting this axis, investigate its role across CNS diseases, and address the challenges of clinical application. By advancing our understanding of neuroinflammatory pathways, these efforts could lead to innovative treatments for AUD and other neurodegenerative conditions. Nonetheless, several questions remain unanswered. For instance, how does NLRP6 activation in astrocytes interact with other CNS immune signaling pathways such as TLRs or RIG-I-like receptors? Is there evidence for feedforward signaling between astrocytes and microglia mediated via extracellular vesicles? Future research should also explore single-cell RNA sequencing datasets from human AUD brains to validate the cellular expression of NLRP6 and miR-339, adding translational weight to these findings.

*We gratefully acknowledge support from the Nebraska Center for Substance Abuse Research (NCSAR).*

*This work was supported by startup funding from UNMC to Dr. PP and partially by the National Institute on Alcohol Abuse and Alcoholism (AA031444 and P50AA030407-5126, Pilot Core grant) to Dr. SS.*
